# Characterisation of digital therapeutic clinical trials: a systematic review with natural language processing

**DOI:** 10.1016/S2589-7500(23)00244-3

**Published:** 2024-03

**Authors:** Brenda Y Miao, Madhumita Sushil, Ava Xu, Michelle Wang, Douglas Arneson, Ellen Berkley, Meera Subash, Rohit Vashisht, Vivek Rudrapatna, Atul J Butte

**Affiliations:** Bakar Computational Health Sciences Institute, University of California, San Francisco, CA, USA; Bakar Computational Health Sciences Institute, University of California, San Francisco, CA, USA; Bakar Computational Health Sciences Institute, University of California, San Francisco, CA, USA; Department of Bioengineering and Therapeutic Sciences, University of California, San Francisco, CA, USA; Bakar Computational Health Sciences Institute, University of California, San Francisco, CA, USA; Bakar Computational Health Sciences Institute, University of California, San Francisco, CA, USA; Department of Clinical Pharmacy, School of Pharmacy, University of California, San Francisco, CA, USA; UC Davis Health, University of California, Sacramento, CA, USA; Division of Rheumatology, Department of Medicine, University of California, San Francisco, CA, USA; School of Biomedical Informatics, UTHealth Houston, Houston, TX, USA; Bakar Computational Health Sciences Institute, University of California, San Francisco, CA, USA; Bakar Computational Health Sciences Institute, University of California, San Francisco, CA, USA; Division of Gastroenterology and Hepatology, Department of Medicine, University of California, San Francisco, CA, USA; Bakar Computational Health Sciences Institute, University of California, San Francisco, CA, USA; University of California Health, Oakland, CA, USA

## Abstract

Digital therapeutics (DTx) are a somewhat novel class of US Food and Drug Administration-regulated software that help patients prevent, manage, or treat disease. Here, we use natural language processing to characterise registered DTx clinical trials and provide insights into the clinical development landscape for these novel therapeutics. We identified 449 DTx clinical trials, initiated or expected to be initiated between 2010 and 2030, from ClinicalTrials.gov using 27 search terms, and available data were analysed, including trial durations, locations, MeSH categories, enrolment, and sponsor types. Topic modelling of eligibility criteria, done with BERTopic, showed that DTx trials frequently exclude patients on the basis of age, comorbidities, pregnancy, language barriers, and digital determinants of health, including smartphone or data plan access. Our comprehensive overview of the DTx development landscape highlights challenges in designing inclusive DTx clinical trials and presents opportunities for clinicians and researchers to address these challenges. Finally, we provide an interactive dashboard for readers to conduct their own analyses.

## Introduction

Digital therapeutics (DTx) are a somewhat novel class of US Food and Drug Administration (FDA)-regulated software that help patients prevent, manage, or treat disease. Beyond providing additional therapeutic options for patients, the method of delivery of DTx also enables the delivery of continuous and personalised care at scale.^1,2^ Examples of approved DTx include the Propeller platform, which uses smart devices and paired consumer applications to improve medication adherence and reduces hospital admissions in patients with asthma and chronic obstructive pulmonary disease (COPD),^3,4^ and EndeavorRx, a video game that helps improve attention function in children with attention-deficit hyperactivity disorder.^5^ Although DTx have the potential to help bridge gaps in access to care, there are concerns that these software will require access to compatible devices or high digital literacy, and widen disparities in health outcomes.^1,6^ There is also substantial interest from health-care and regulatory institutions to analyse the clinical development landscape and quality of clinical evidence available for DTx.^6,7^

ClinicalTrials.gov is the main website in the USA for registering clinical trials, as required by the FDA Amendments Act of 2007.^8^ Several studies have previously used the ClinicalTrials.gov registry to characterise the level of clinical evidence for drug therapeutics, including analysis of clinical trial design and applicability of trial results to real-world populations. ^9–11^ Analogous studies of clinical trials involving digital interventions^12–14^ have focused on structured data fields, and only a few have attempted to provide additional insights through manual free-text analysis. However, manual analysis is time-consuming, requires specialised expertise, and is difficult to keep up to date as new DTx trials occur, and so automated tools are necessary to provide real-time insight into emerging trials.

In the past 5 years, developments in natural language processing (NLP) have made automated information extraction readily available for biomedical text. Software tools, such as SciSpacy, provide open-source access to text analysis pipelines and NLP models, which are pretrained on large biomedical datasets and can achieve high accuracies on information extraction and other language tasks.^15,16^ These pipelines can also map extracted concepts to existing biomedical vocabularies, such as MeSH categories, for standardisation and downstream analysis. Several NLP methods have been applied to analyse drug therapeutic clinical trials,^11,17^ but have not yet been used to characterise the clinical development of DTx.

Given the increasing availability of DTx and their corresponding clinical trials, we did a systematic review to describe the characteristics of trials on DTx. We took advantage of modern NLP methods to better understand the characteristics of DTx clinical trials and the quality of evidence available for these novel therapeutics. Finally, we provide an interactive dashboard for readers to do their own analyses of DTx studies using structured and unstructured data fields from ClinicalTrials.gov.

## Methods

### Search strategy and selection criteria

Digital therapeutics clinical trials were identified through the ClinicalTrials.gov application programming interface by use of a set of 27 search terms related to DTx, including “digital therapeutic”, “digital therapy”, “smartphone”, “mobile app”, and “video game” (appendix p 1). Searches were limited to the fields for BriefSummary, BriefTitle, InterventionName, InterventionDescription, Keyword, DetailedDescription, EligibilityCriteria, or OfficialTitle, and only trials registered for FDA-regulated devices and not listed as having a “basic science purpose” were included. We used the ClinicalTrials.gov field IsFDARegulatedDevice to identify trials “studying a device product subject to section 510(k), 515, or 520(m) of the Federal Food, Drug, and Cosmetic Act”.^18^ Thus, even if FDA clearance or approval had not been granted for any of these trials, there was a high degree of confidence that they were for FDA-regulated products. Basic science studies were identified with the DesignStudyPurpose field and were removed to focus on trials of DTx with an established mechanism of action. By use of the OverallStatus field, trials that had been terminated, withdrawn, suspended, or had an unknown status were also excluded to limit analysis to active trials. The scope of the systematic review was also limited to studies with start dates occurring after 2010, or expected completion dates listed after 2030. Following these filtering steps, the full record from each remaining DTx trial was then extracted from the complete ClinicalTrials.gov dataset, which was downloaded on Aug 3, 2022. We report our findings in line with PRISMA guidelines. Since this systematic review does not assess health outcomes, no protocol is registered on PROSPERO. The full list of data fields available for each trial can be found on ClinicalTrials.gov on the Protocol Registration Data Element Definitions page.^18^

### Analysis of clinical trial characteristics by use of structured data fields

We compared the number and duration of interventional and observational trials, with duration calculated as the number of years between reported start and completion dates. Clinical trials were also analysed on the basis of sponsor and collaborator types, visualised with a Sankey diagram. To understand the geographical distribution of clinical trial facilities in the USA, each entry in the LocationState field was mapped to a state code with the pgeocode software package (version 0.3.0) and the number of trials in each state was plotted as a choropleth map. The density of clinical trial facilities in each state was also calculated as a ratio of trial locations to the population of each state, by use of the 2021 estimated US Census Bureau values.^19^

We analysed correlation between the number of clinical trial locations and the area deprivation index (ADI), a metric of socioeconomic status in each region. ADIs for the five states with the highest number of clinical trial locations were downloaded from the University of Madison Neighborhood Atlas and mapped to each listed facility’s zip code.^20,21^ National and state ADIs were analysed, with national ADI score given as a percentile across the entire country. At the state level, ADI is provided on a scale from 1 to 10. Higher scores represent greater socioeconomic disadvantage for both state and national ADIs. Only trials with available features in each data field were considered for these analyses (appendix p 2).

### Extraction of condition and eligibility criteria by use of NLP

Although ClinicalTrials.gov has an internal algorithm to map conditions listed with standardised biomedical vocabulary to MeSH terms, these terms do not correspond to the main MeSH branches and are not available for all clinical trials.^22^ To create standardised mappings for each clinical trial, medical conditions from the condition free-text field were extracted and mapped to MeSH terms by use of the MeSH EntityLinker from SciSpacy (version 0.5.0),^15^ with only the first match selected for each condition. Resulting terms were grouped into MeSH categories and the most frequent heading was selected for trials with multiple conditions, with priority given to values under the branches C (diseases) and F (psychiatry and psychology). MeSH terms were manually reviewed to assess the validity of the MeSH EntityLinker on this dataset. With conditions classified into standardised clusters, we compared enrolment counts in each MeSH heading, focusing on non-phase 1, interventional trials in groups with fewer than ten studies. The EnrolmentType field was used to differentiate between actual and anticipated enrolment for each trial.

To analyse the most common types of eligibility criteria, we used the BERTopic topic modelling technique (version 0.11.0),^23^ which clusters text embeddings to produce interpretable, semantically cohesive clusters. BERTopic has been used in previous studies of biomedical text and has been shown to generate more coherent topics compared with Latent Derelict Aldrich or other topic modelling methods.^24^ To generate embeddings for BERTopic, text from the eligibility criteria field was first split into inclusion and exclusion criteria, with each line con sidered a separate document. A language model from SciSpacy pretrained on biomedical text (en_core_sci_lg) was then used to generate embeddings for each eligibility criterion. The SciSpacy embeddings encode semantic relationships between biomedical terms, allowing related terms to be grouped into more semantically cohesive topics, unlike conventional methods that cluster words only on the basis of their frequency and co-occurence.^15^ A BERTopic model with default settings was used to generate topics from these embeddings, and the top five topics for each eligibility criterion were mapped back to the corresponding clinical trial to analyse the percentage of each topic occurring in each MeSH cluster. Again, a subset of the 200 inclusion and exclusion criteria were manually reviewed to confirm that the eligibility criteria were mapped correctly to these topics. Only MeSH groups with at least 15 studies were analysed. Topic modelling was done on inclusion and exclusion criteria of interventional trials in our dataset not listed as a phase 1–4 trial.

### Development of an interactive dashboard for DTx clinical trial analysis

The dashboard for clinical trials data analysis was built with Streamlit. The dashboard implements all the methods described in this systematic review for analysis of study types, sponsor types, conditions, and eligibility criteria.

### Statistics

Descriptive statistics are provided for categorical variables as proportions, and averages are reported for continuous variables as medians and IQRs. Spearman’s rank correlation coefficient (*r*) values were calculated to analyse the correlation between continuous variables. Mann-Whitney *U* tests were used to establish differences in median enrolment between MeSH categories and Bonferroni correction was used to account for multiple testing. Statistical testing was done with Scipy (version 1.7.3) and p values less than 0·05 were considered significant.^25^

## Results

**Figure 1:**
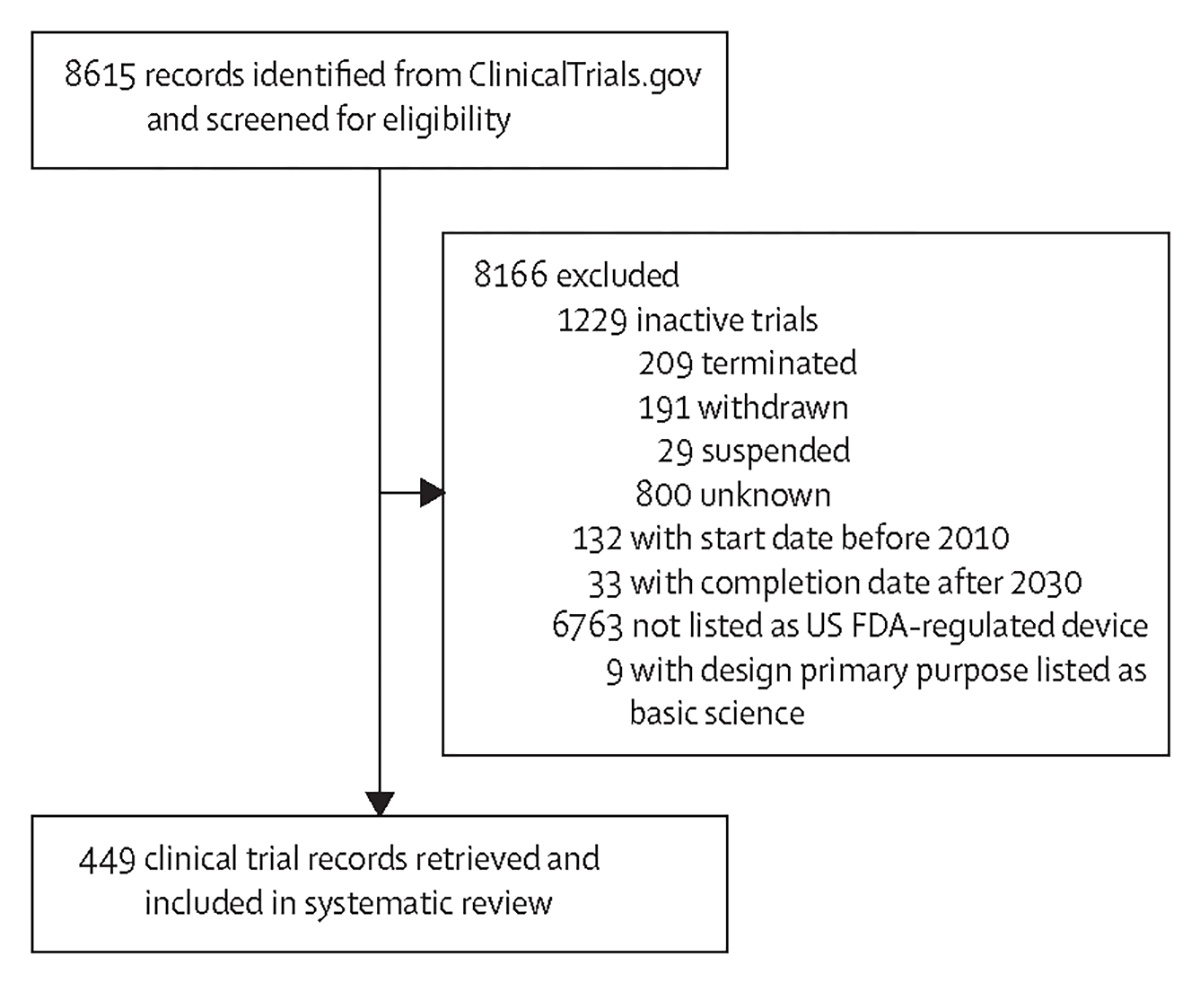
Study selection Identification of 449 DTx clinical trial datasets from a search of ClinicalTrials.gov by use of 27 search terms and additional ClinicalTrials.gov data filters. DTx=digital therapeutics. FDA=US Food and Drug Administration.

**Figure 2:**
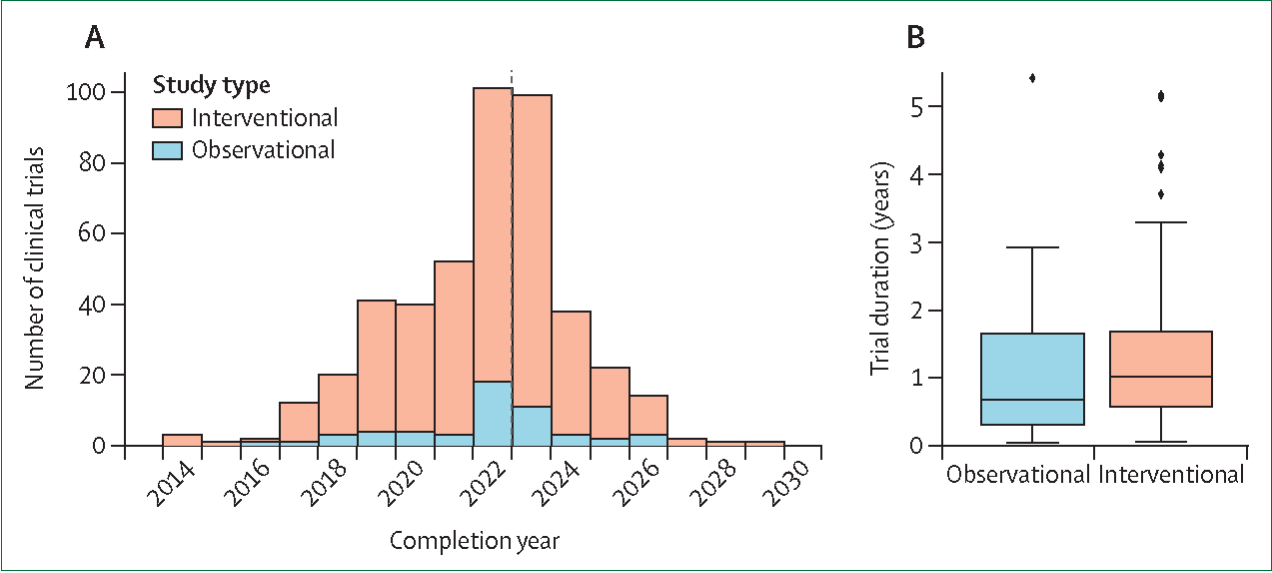
Overview of DTx clinical trials (A) Number of trials completed or expected to complete between 2014 and 2030. The dashed line indicates the current year. (B) Duration of completed interventional and observational trials. DTx=digital therapeutics.

Using 27 search terms related to digital therapeutics (appendix p 1), we identified 8615 clinical trials involving digital-based interventions. Of these trials, 7386 were active or ongoing, and 7221 had a start date after 2010 and expected completion date before 2030. Since DTx are regulated by the FDA as “software as a medical device”, we only considered studies that were listed as using FDA-regulated devices and conducted for non-basic science purposes, resulting in 449 studies of interest (figure 1). Of these 449 studies, 53 (11∙8%) were observational and 396 (88∙2%) interventional (figure 2), with 74 interventional studies listing a completion date in 2022, and 88 in 2023. Overall, 150 interventional and 18 observational studies were listed as completed, with median study durations of 1·02 years (IQR 0·57–1·69, range 0·06–5·17) and 0·69 years (0·32–1·59, range 0·05–5·42), respectively (figure 2). 13 observational and 68 interventional studies were first posted to the registry in 2022 (appendix p 3). Because all information on ClinicalTrials.gov is voluntarily reported by the sponsor of each clinical trial, only available data are used for each analysis and missingness is reported in the appendix (p 2).

ClinicalTrials.gov requires sponsors to list the facilities in which studies are being done, although how this is interpreted for DTx studies is not clear. As one of the primary advantages of DTx is their ability to deliver care remotely, we wanted to understand the geographical distribution of listed physical clinical trial locations.

**Figure 3:**
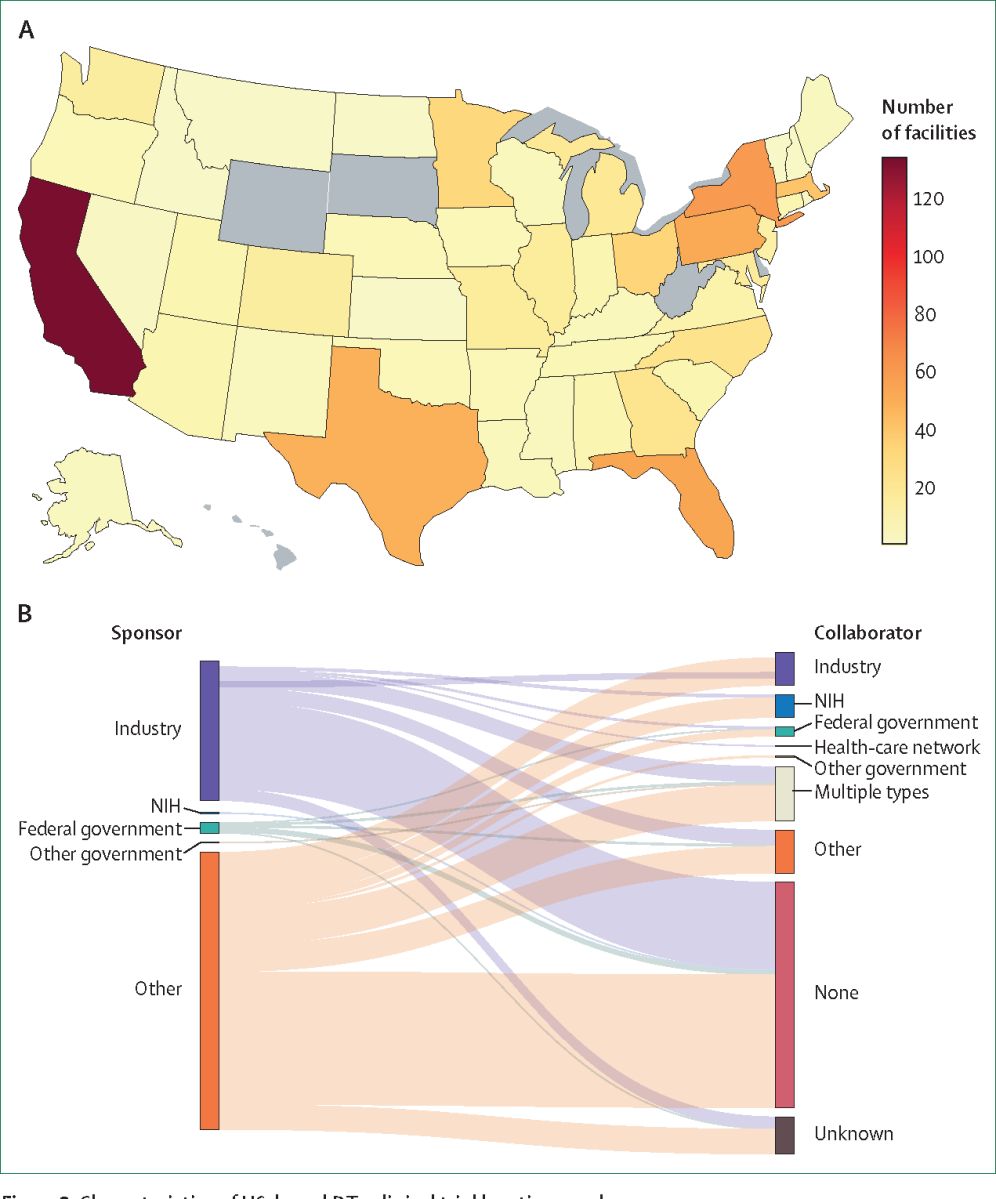
Characteristics of US-based DTx clinical trial locations and sponsors (A) Number of facilities doing DTx clinical trials by state. Grey areas represent states with no clinical trials. (B) Distribution of sponsor and collaborator types. DTx=digital therapeutics. NIH=National Institutes of Health.

Using location data provided by each study, we found that the states with the most DTx clinical trial locations were California (n=135), New York (n=58), Florida (n=55), Pennsylvania (n=52), and Texas (n=50; figure 3). Five states—South Dakota, Wyoming, Hawaii, Delaware, and West Virginia—had no listed locations. Overall, the mean number of locations for each completed trial was 2·33 (SD 5·75). Four trials were completed without any listed facilities. The number of clinical trial locations was strongly correlated with state population (*r*=0·89, p<0·001; appendix p 4). We also analysed whether the reported clinical trial locations included socioeconomically disadvantaged neighbour hoods, measured with the ADI. In the five states with the largest number of clinical trial locations, the number of clinical trials was inversely correlated with both the national (*r*=−0·52, p<0·001) and state (*r*=−0·66, p=0·037) ADI (appendix p 4).

To characterise the types of sponsors and collaborators funding or supporting clinical trials for DTx, we looked at the listed lead sponsor and collaborator classes for the 449 trials. The most common sponsor type was other (n=290 [65%]), which generally referred to academic medical centres (figure 3). Industry was the next most common sponsor type, with 146 (33%) trials. Most studies were done by a single sponsor with no collaborators (n=236, 53%), 131 (29%) had one collaborator, 45 (10%) had two, and 37 (8%) had three or more. For studies with a single collaborator, 26 were sponsored by other or academic institutions and had an industry collaborator and 14 were sponsored by industry with another or academic collaborator.

**Figure 4:**
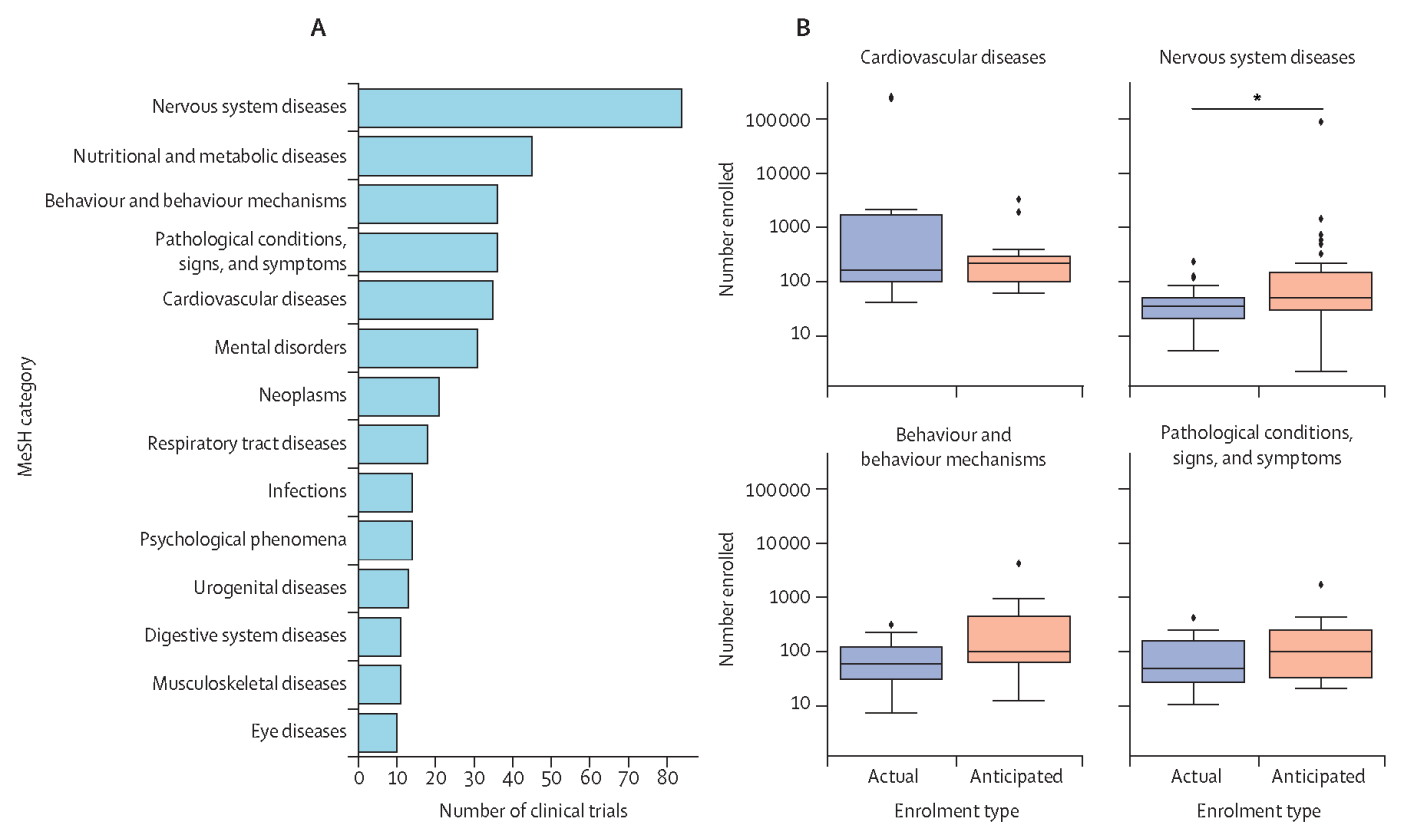
Interventional DTx clinical trials by medical specialty (A) Number of clinical trials mapped to each MeSH term by use of a SciSpacy EntityLinker.^15^ (B) Actual and anticipated enrolment by MeSH group. Diamonds represent outliers. DTx=digital therapeutics. *Significant (p<0·05)

To establish the distribution of DTx trials by medical specialty, we mapped conditions listed as free text by each clinical trial to MeSH terms using a SciSpacy pipeline and MeSH EntityLinker. The three most common headings tested in DTx clinical trials were nervous system diseases (n=82 [19%]; figure 4), nutritional and metabolic diseases (n=45 [10%]), and pathological conditions, signs, and symptoms (n=41 [9%]), followed by behaviour and behaviour mechanisms (n=37 [8%]), cardiovascular diseases (n=34 [8%]), and mental disorders (n=31 [7%]). Conditions that mapped to the heading of nervous system diseases included stroke and Parkinson’s disease, nutritional and metabolic diseases included both diabetes type 1 and 2, and respiratory tract diseases included conditions such as asthma and COPD. The MeSH category pathological conditions, signs, and symptoms contained “abnormal anatomical or physiological conditions…not classified as disease”, and included conditions such as chronic pain. Manual review of MeSH terms also showed that this approach mapped conditions to appropriate categories for 95% of conditions (appendix pp 5–6). Of the six studies in which conditions did not map to MeSH terms and were excluded from analysis, four described treatments or device characteristics (eg, device latency) rather than medical conditions and two described generic symptoms that did not map to specific headings (nasal congestion and prenatal stress; appendix p 7).

With conditions classified into standardised clusters, we compared enrolment counts within each MeSH heading, focusing on non-phase 1, interventional trials in groups with fewer than ten studies. Trials targeting cardiovascular diseases had the highest number of actual and anticipated participants, with a combined median of 200 participants (IQR 100–350, range 40–450 000; 24 trials), followed by trials for nutritional and metabolic diseases with a combined median of 100 participants (IQR 30–197, range 6–6006; 41 trials) and behaviour and behaviour mechanisms again with a combined median of 100 participants (IQR 40–234, range 7–4500; 35 trials; figure 4). The category with the fewest median number of participants was nervous system diseases, which had a median of 40 participants (IQR 22–100; 70 trials), although the largest trial in this category listed an anticipated enrolment of 100 000 participants. Comparing anticipated and actual enrolment information within each MeSH group, median anticipated enrolment was only significantly higher than actual enrolment for nutritional and metabolic disease DTx trials, with a median difference of 211 participants (p=0·035).

**Figure 5:**
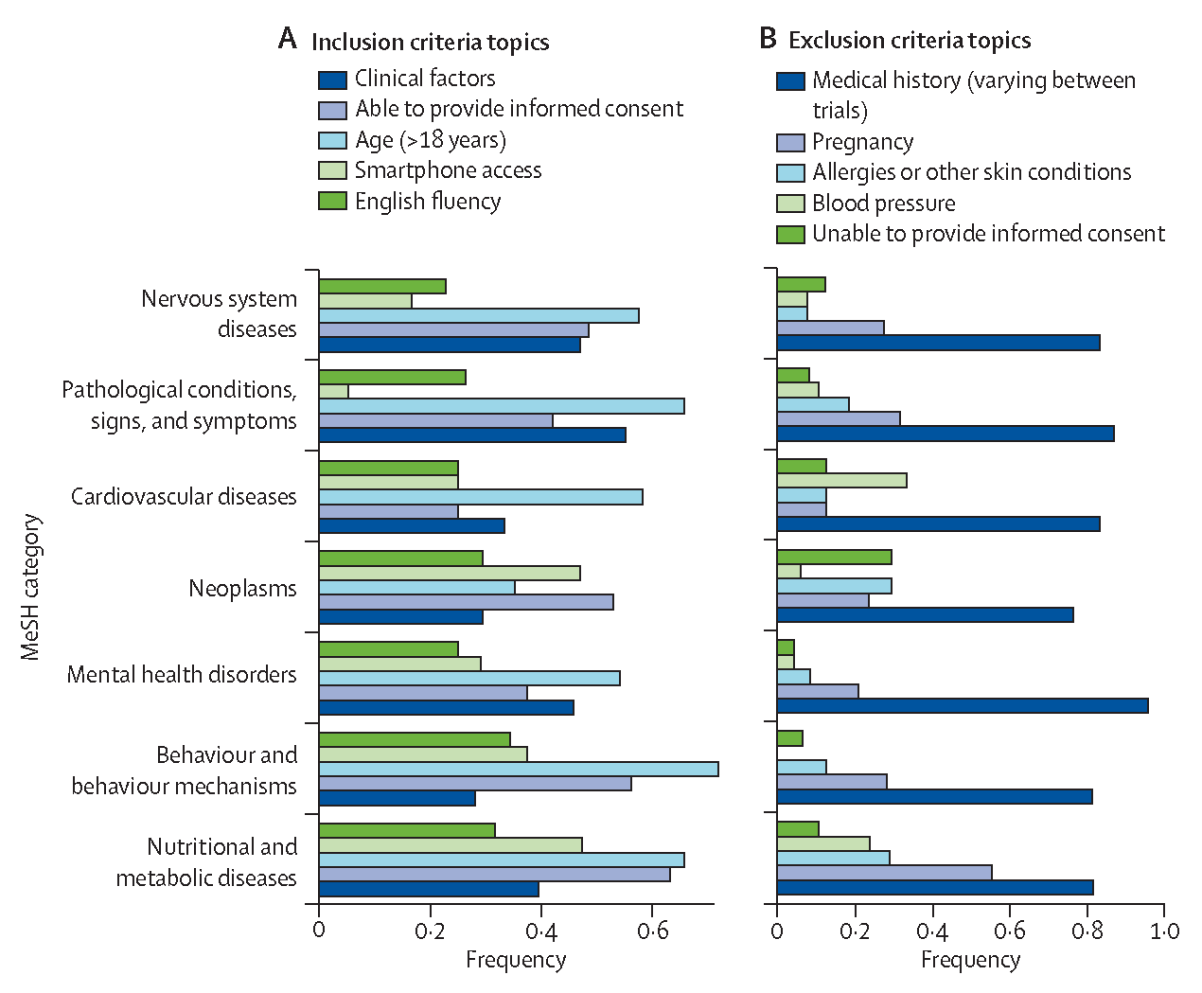
Topic analysis of DTx clinical trial eligibility BERTopic embedding clustering was used for topic modelling of inclusion (A) and exclusion (B) criteria of DTx trials within each MeSH term. DTx=digital therapeutics.

Previous studies of drug therapeutic clinical trials have shown that eligibility criteria are often overly strict and can skew trial cohorts away from real-world patient populations.^10,11^ The top five inclusion criteria topics identified by BERTopic from DTx studies were defined by terms related to clinical factors, ability to provide informed consent, age, smartphone and data access, and English fluency (figure 5). Criteria associated with clinical factors were most frequently found in 21 (55%) of 38 pathological condition trials, 31 (47%) of 66 trials for nervous system diseases, and 11 (46%) of 24 trials for mental health disorders. Age criteria were most likely to be found in trials for behavioural disorders (23 [72%] of 32) and nutritional and metabolic diseases (25 [66%] of 38). Inclusion criteria detailing smartphone access were also found in several trials, occurring most frequently in DTx intended for nutritional and metabolic diseases (18 [47%] of 38) and neoplasms (8 [47%] of 17), and least frequently in trials for nervous system diseases (11 [17%] of 66) and pathological conditions (2 [5%] of 38). The topic related to smartphones and data access also contained other keywords associated with device compatibility, cellular data plans, and Wi-Fi access. Manual review of DTx studies with eligibility criteria in this topic showed that patients could be excluded if they did not have a PayPal account (NCT04857515), were not willing to use a smartphone and personal data plan, (NCT04159480), or did not show technological literacy (NCT04136626). This topic was most frequently found in trials for nutritional and metabolic diseases (18 [47%] of 38). The ability to provide informed consent was also most frequently found in trials for nutritional and metabolic diseases (24 [63%] of 38) and English fluency criteria occurred most frequently in trials for behaviour and behaviour mechanisms (11 [34%] of 32).

The top topics generated from the exclusion criteria were associated with medical history (varying between trials), pregnancy, allergies or other skin conditions, blood pressure, and, as with the inclusion criteria, the ability to provide informed consent (figure 5). 23 (96%) of 24 DTx clinical trials targeting mental health disorders, 33 (87%) of 38 trials targeting nervous system diseases, and 20 (83%) of 24 trials targeting cardiovascular disease had exclusion criteria associated with medical history. Component analysis showed that some trials specifically excluded patients with a history of smoking or suicidal behaviour, cardiac disorders, or use of insulin (appendix p 9). Analysis of the topic associated with pregnancy showed that nutritional and metabolic disease DTx trials were most likely to contain this exclusion criterion (21 [55%] of 38), but only five (21%) of 24 trials for mental health disorders and three (13%) of 24 trials for cardiovascular diseases listed such criteria. Manual review was done on a subset of inclusion and exclusion eligibility criteria to ensure that topics were highly coherent and accurately described each criterion. Topics were appropriate in 95% (n=200) of inclusion criteria and 94% (n=200) of exclusion criteria (appendix pp 10–11).

Although ClinicalTrials.gov has filters and other data analysis tools that enable research into the structured data, there are few publicly available visual tools for the analysis of DTx clinical trials. We provide an interactive dashboard—available from Github—for the analysis of DTx clinical trials data by use of the methods described in this Review.

## Discussion

Digital therapeutics are a unique method of delivery for treating disease and have the potential to provide new treatment options for patients at an unprecedented scale. Here, we used NLP pipelines to characterise 449 DTx clinical trials identified on ClinicalTrials.gov. With more than 150 of these trials having expected completion dates by 2023, DTx are becoming rapidly available for patient care, making it essential to characterise the quality of evidence being gathered for these novel therapeutics and to better understand their benefits for real-world patient populations.

We showed that the majority of DTx trials are sponsored by academic institutions or industry with no collaborators and are primarily being developed for nervous system diseases and nutritional and metabolic diseases, which aligns with a previous review of DTx clinical trials.^14^ However, the review relied on manual extraction of DTx and did not filter for FDA-regulated devices with the ClinicalTrial.gov data field. Although we were able to quantify the distribution of sponsor categories, this study did not investigate any funding sources for these sponsors or the cost of DTx trials. ClinicalTrials.gov does provide an optional field for sponsors to include information regarding grants and funding sources, but its completeness and accuracy is dependent on transparent reporting from sponsors, and future studies might be necessary to quantify funding and costs for these trials.

Our results also indicated that DTx trials were often of short duration, with interventional studies lasting an average of only 1 year, which points to a need for additional studies to understand the long-term usage and efficacy of DTx. Although these trials are short, the largest DTx trials were able to enrol more than 400 000 patients in only one or two locations, suggesting that either these trials can be effectively scaled, or that they have alternative patient recruitment strategies that ClinicalTrials.gov does not capture. However, we also showed that DTx clinical trial facilities tend to be in the most populated states. Few are done in socioeconomically disadvantaged neighbourhoods, but further research is necessary to understand the true geographical and demographic distributions of users.

Analysis of DTx clinical trial eligibility criteria showed that these trials frequently exclude patients with comorbidities, who are pregnant, who are children, and who are not fluent in English. Eligibility criteria for drug therapeutics frequently cause clinical trial cohorts to deviate from real-world populations,^10,11^ and analogous research into DTx usage might be necessary to ensure trial results are applicable to general patient populations. We also identified criteria specific to digital determinants of health, which describe factors related to the accessibility or availability of technology that contribute to health outcomes and quality of life.^26,27^ Our geographical analysis of these studies also matched this finding, which suggested that fewer facilities in disadvantaged communities in the USA are being used to recruit participants. Future initiatives to assess the role of digital determinants of health, such as SOLVE Health Tech,^28^ are necessary to ensure that DTx are effective in promoting better outcomes for all patients.

The insights here and in the online interactive dashboard provide a framework for future research into DTx clinical trials, although we recognise there are limitations to our study. Although we were stringent in limiting our analysis to only FDA-regulated DTx, we might have missed DTx regulated outside the USA or inadvertently removed or selected others with our search criteria. Some DTx cleared through the 510(K) pathway, which allows medical devices to be marketed if they are substantially equivalent to already cleared devices, might not have registered preapproval trials,^6^ but might still require post-marketing trials that could be analysed in future studies. Additionally, we were not able to differentiate between safety and efficacy studies with the data fields provided by ClinicalTrials.gov. Our analysis is also inherently limited to sponsor-provided data, which are not always up to date or accurate and might be missing or unstandardised.^22^ These limitations are particularly true for observational studies, for which the investigators are not required to list if they are studying an FDA-regulated product or if they accept healthy volunteers,^18^ although requirements could change as regulatory pathways evolve for the use of real-world evidence in clinical trials. Finally, we focused on the use of MeSH terminology in our pipelines due to the suggested use of such terminology on ClinicalTrials.gov, but other clinical vocabularies might be more applicable to capture additional nuances in clinical trial metadata analyses. Although we took a conservative approach in mapping DTx clinical trials to broad MeSH terms, clinical trials might also involve different indications that could be better captured by allowing trials to be mapped to multiple MeSH categories.

Despite the limitations, our application of NLP strategies to ClinicalTrials.gov provides a comprehensive overview of the DTx development landscape, and the modular dashboard developed here will serve as an openly available tool for future research into clinical trial design and the real-world applicability of DTx.

## Supplementary Material

Supp data

## Data Availability

Data, source code, and a link to the DTx clinical trial analysis dashboard are available via Github. Options are available to filter the data by different study fields, and the processed dataset, including results from NLP methods, can be downloaded from Github for further analysis.
