# Supplementary material for: Characterisation of digital therapeutic clinical trials: a systematic review with natural language processing
Source: Lancet Digit Health. Author manuscript; Available in PMC 2024 May 7. (PMC11074920; doi:10.1016/S2589-7500(23)00244-3)
Supplement: Supp data [file NIHMS1979894-supplement-Supp_data.pdf]

THE LANCET
Digital Health

Supplementary appendix

This appendix formed part of the original submission and has been peer reviewed. We post it as supplied by the authors.

Supplement to: Miao BY, Sushil M, Xu A, et al. Characterisation of digital therapeutic clinical trials: a systematic review with natural language processing. *Lancet Digit Health* 2024; **6:** e222–29.

Contents of supplementary figures and tables

Table S1..1
Figure S1..2
Figure S2..3
Figure S3..4
Table S2..5
Table S3..7
Table S4..8
Table S5..9
Table S6..10
Table S7..11
Figure S4..12

Supplementary figures

Table S1. Number of search results returned from ClinicalTrials.gov for each DTx-associated term.

Search term	Number of studies
"digital therapeutic"	76
"digital therapy"	33
"digital therapies"	33
"mobile health"	1598
"smartphone"	3219
"smart phone"	3219
"digital intervention"	156
"mobile platform"	53
"mobile app"	1834
"mobile device"	462
"study app"	48
"digital treatment"	27
"android"	401
" app."	1265
" app,"	766
"digital tablet"	21
" ios"	340
"iphone"	219
"smart watch"	83
"smartwatch"	140
"virtual reality"	1747
"video game"	598
"digital health"	388
"mobile video"	12
"digital platform"	120
"software intervention"	7
"software treatment"	8

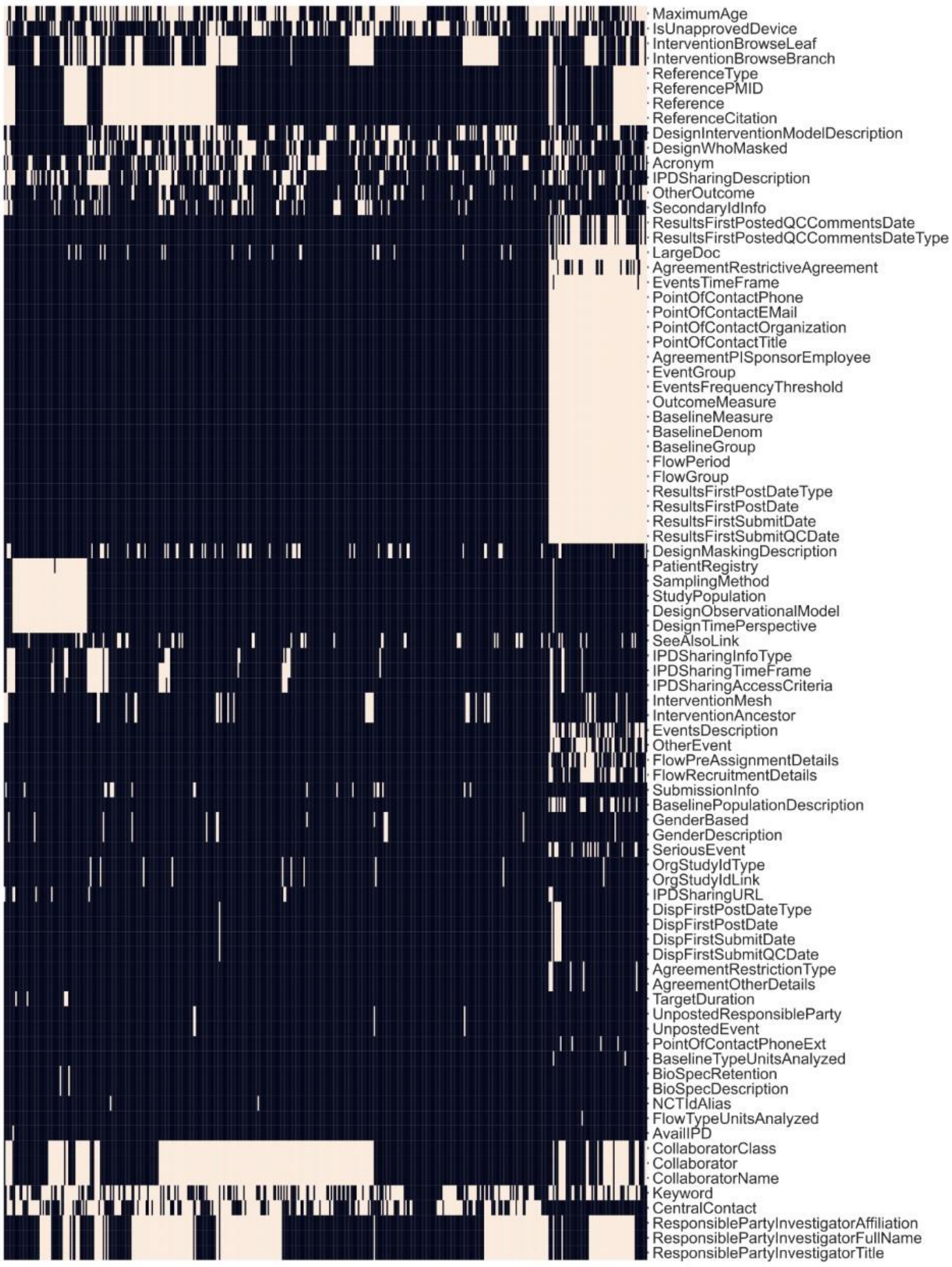

Figure S1. Missing values in DTx clinical trials for each data field. Only data fields where at least 1 trial contains a missing value is shown. Black bars indicate missing data.

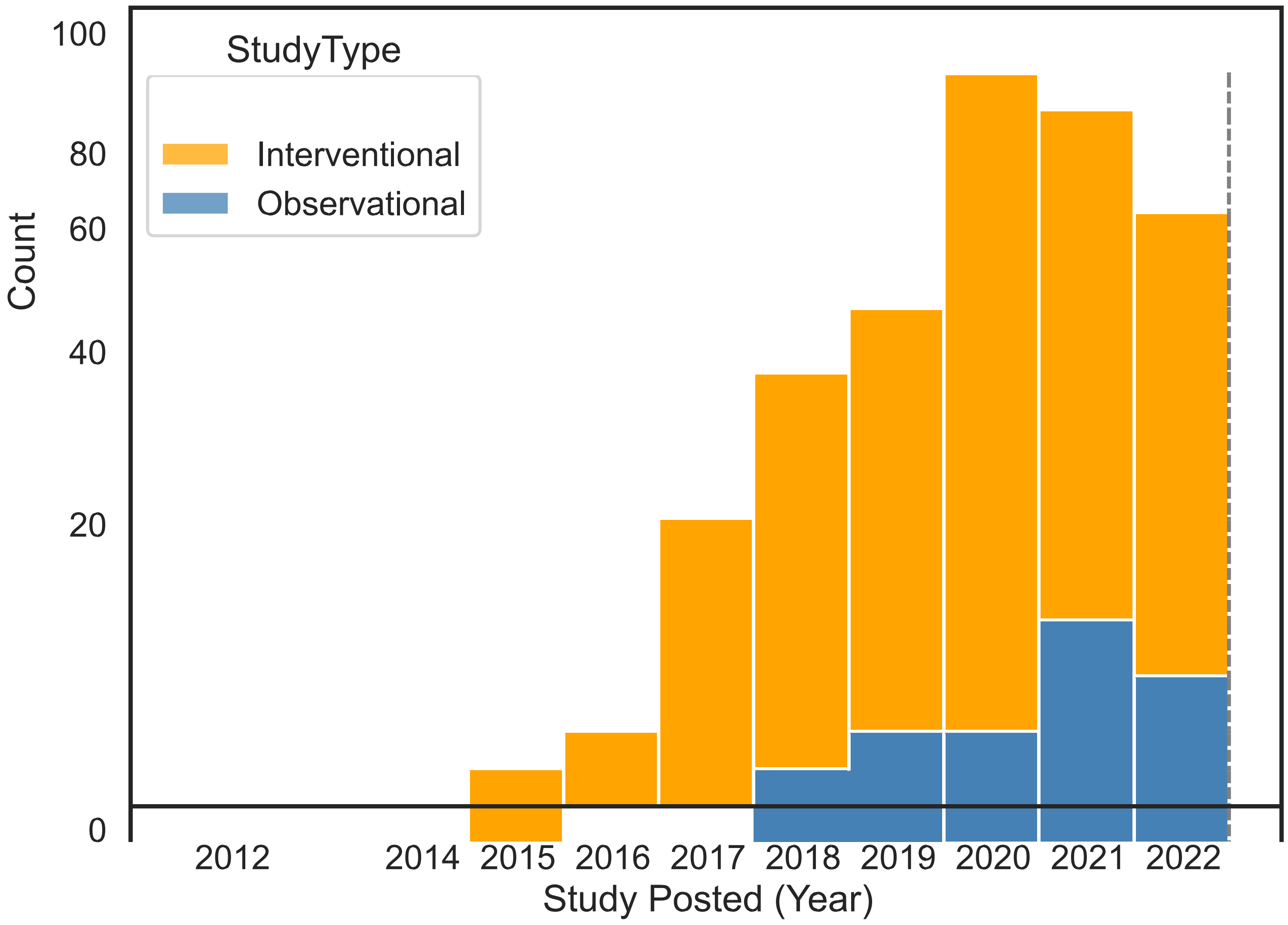

Figure S2. Distribution of study post dates for digital therapeutic clinical trials.

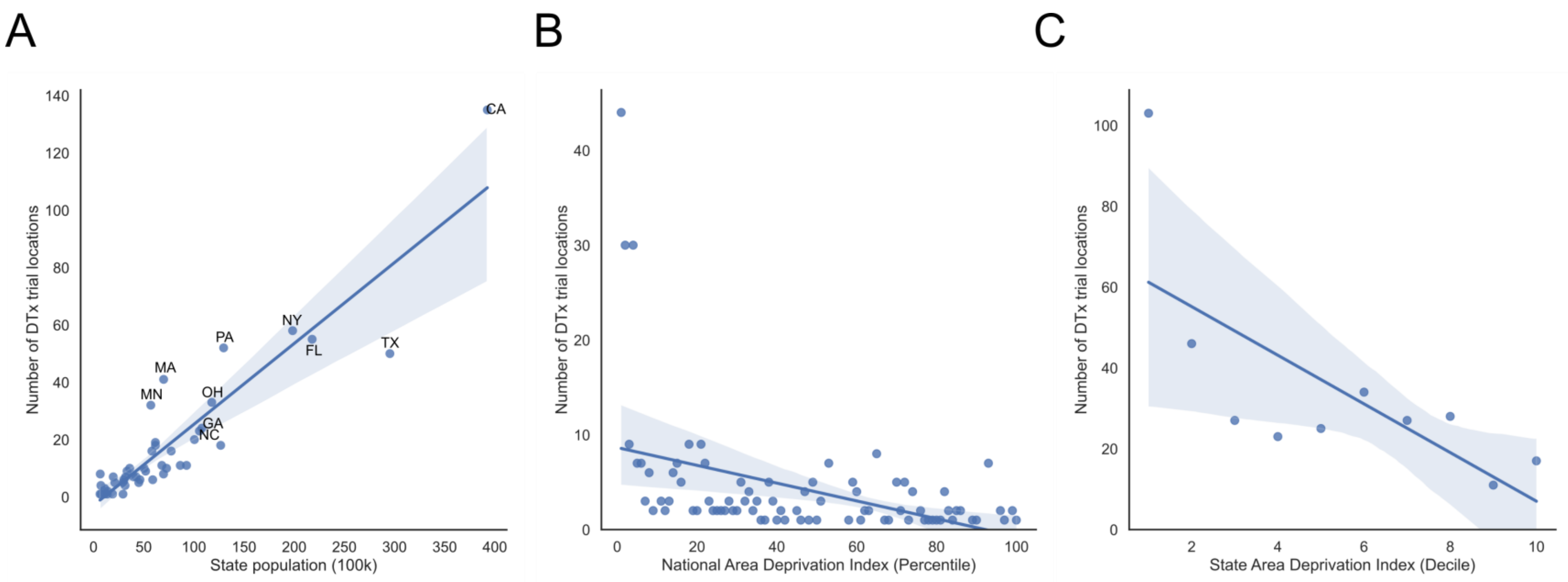

Figure S3: Correlation between number of DTx clinical trial locations and geographic characteristics. A) Correlation between number of DTx clinical trials and state population. The top 10 states with the highest number of DTx clinical trials are labeled. Within the top 5 states, we also looked at the relationship between number of DTx trial locations and area deprivation index (ADI) at the B) national level and C) state level. The national ADI is calculated relative to other zip codes in the United States, while the state ADI is relative to other zip codes in the state. Higher ADI scores at both the national and state level indicate greater socioeconomic disadvantage.

Table S2: Incorrect MeSH branches selected by SciSpacy EntityLinker.

Clinical Trial	Condition (ClinicalTrials.gov)	MeSH branch (SciSpacy EntityLinker)	Incorrect ("N") or "Multiple" possible MeSH Branches
**NCT03934658**	['PostTraumatic Stress Disorder' 'Sleep Disorder' 'Stress Disorder' 'Sleep Initiation and Maintenance Disorders' 'Combat Disorders' 'Nightmares Associated With Chronic Post-Traumatic Stress Disorder' 'Nightmare' 'Nightmares, REM-Sleep Type']	Psychological Phenomena	N
**NCT03828656**	['PostTraumatic Stress Disorder' 'Sleep Disorder' 'Stress Disorder' 'Sleep Initiation and Maintenance Disorders' 'Combat Disorders' 'Nightmares Associated With Chronic Post-Traumatic Stress Disorder' 'Nightmare' 'Nightmares, REM-Sleep Type']	Psychological Phenomena	N
**NCT03795987**	['Stress Disorders, Post-Traumatic' 'Combat Disorders' 'Ptsd' 'Nightmare' 'Nightmares, REM-Sleep Type' 'Nightmare Disorder With Associated Non-Sleep Disorder']	Psychological Phenomena	N
**NCT04040387**	['Stress Disorders, Post-Traumatic' 'Combat Disorders' 'Ptsd' 'Nightmare' 'Nightmares, REM-Sleep Type' 'Nightmare Disorder With Associated Non-Sleep Disorder']	Psychological Phenomena	N
**NCT04897074**	['Attention Deficit Hyperactivity Disorder']	Psychological Phenomena	N
**NCT04418076**	['HIV/AIDS' 'Cocaine Use']	Organic Chemicals	N
**NCT04846777**	['Generalized Anxiety Disorder']	Investigative Techniques	N
**NCT03748264**	['Sleep Disordered Breathing']	Psychological Phenomena	N
**NCT05077644**	['Post-partum Depression']	Urogenital Diseases	N
**NCT04364256**	['Autologous Hematopoietic Stem Cell Transplant']	Biological Factors	N
**NCT04684823**	['Cystic Fibrosis' 'Adherence, Medication']	Digestive System Diseases	N
**NCT03047720**	['Nocturnal Enuresis']	Heterocyclic Compounds	N
**NCT03649074**	['Attention Deficit Hyperactivity Disorder']	Psychological Phenomena	N
**NCT03678402**	['High Risk for Falling']	Investigative Techniques	N
**NCT04429009**	['Thoracic Surgery' 'Respiratory Therapy']	Health Occupations	N
**NCT05147987**	['Insufficient Lactation']	Urogenital Diseases	N
**NCT05454813**	['System Validation']	Hemic and Immune Systems	N
**NCT04584970**	['Scoliosis Idiopathic' 'Pain, Postoperative']	Infections	N
**NCT05150197**	['Visual Field Defect, Peripheral']	Diagnosis	N
**NCT04416555**	['Postoperative Pain']	Therapeutics	N
**NCT04268901**	['Phlebotomy' 'Orthopedics' 'Radiology' 'Pain' 'Anxiety' 'Virtual Reality' 'Allergy' 'Gastroenterology']	Immune System Diseases	N
**NCT04175444**	['Visual Field Defect, Peripheral']	Diagnosis	N
**NCT04025814**	['Attention Deficit Hyperactivity Disorder']	Psychological Phenomena	N
**NCT04857515**	['Smoking Cessation' 'Smoking Behaviors' 'Smoking Reduction' 'Smoking, Cigarette' 'Smoking' 'Nicotine Dependence']	Behavior and Behavior Mechanisms	Multiple
**NCT05365607**	['Posttraumatic Stress Disorder' 'Cardiovascular Diseases' 'Autonomic Dysfunction' 'Vascular Stiffness' 'Nightmare' 'Endothelial Dysfunction']	Mental Disorders	Multiple
**NCT03340311**	['Gestational Diabetes Mellitus']	Urogenital Diseases	Multiple
**NCT04808609**	['Smoking Cessation' 'Smoking' 'Smoking Behaviors' 'Smoking Reduction' 'Smoking, Tobacco' 'Smoking, Cigarette' 'Hiv' 'HIV/AIDS']	Behavior and Behavior Mechanisms	Multiple
**NCT04609514**	['HIV/AIDS' 'Smoking Cessation' 'Tobacco Use Disorder']	Behavior and Behavior Mechanisms	Multiple
**NCT04854798**	['Covid19' 'Cytokine Storm' 'Inflammation']	Pathological Conditions, Signs and Symptoms	Multiple
**NCT04701489**	['Covid19' 'Cytokine Storm' 'Inflammation']	Pathological Conditions, Signs and Symptoms	Multiple
**NCT04838925**	['Chronic Pain' 'Opioid Use']	Pathological Conditions, Signs and Symptoms	Multiple
**NCT04217551**	['Cardiac Arrest, Out-Of-Hospital' 'Hypothermia, Induced' 'Hypoxia-Ischemia, Brain']	Pathological Conditions, Signs and Symptoms	Multiple
**NCT04332718**	['Stroke' 'Atrial Fibrillation']	Nervous System Diseases	Multiple
**NCT03519451**	['Depression' 'Tobacco Use Disorder' 'Current Every Day Smoker']	Behavior and Behavior Mechanisms	Multiple
**NCT03475147**	['Scotoma']	Nervous System Diseases	Multiple
**NCT04465682**	['Urine Detectable Acute and Chronic Diseases']	Pathological Conditions, Signs and Symptoms	Multiple
**NCT04297969**	['Amblyopia Bilateral' 'Hyperopia of Both Eyes' 'Astigmatism Bilateral' 'Accommodation Disorder']	Nervous System Diseases	Multiple
**NCT04607460**	['Chronic Low-back Pain' 'Mastectomy' 'Lumpectomy' 'Migraine']	Nervous System Diseases	Multiple
**NCT04659564**	['Breast Cancer Related Lymphedema' 'Lymphedema of Upper Arm' 'Lymphedema' 'Quality of Life']	Hemic and Lymphatic Diseases	Multiple

**NCT03506568**	['Medication Adherence' 'Glaucoma']	Behavior and Behavior Mechanisms	Multiple
**NCT04205370**	['Sleep' 'Pregnancy Complications']	Psychological Phenomena	Multiple
**NCT04721067**	['Hiv' 'Insomnia']	Nervous System Diseases	Multiple
**NCT05212129**	['Functional Gastrointestinal Disorders' 'Hypermobile Ehlers-Danlos Syndrome' 'Postural Orthostatic Tachycardia Syndrome' 'Autonomic Nervous System Disease' 'Autonomic Nervous System Imbalance']	Nervous System Diseases	Multiple
**NCT05099874**	['Sickle Cell Disease' 'Attention Deficit' 'Cognitive Deficit in Attention']	Hemic and Lymphatic Diseases	Multiple
**NCT05427734**	['Suicide' 'Suicide, Attempted' 'Suicidal Ideation' 'Alcohol Use Disorder' 'Alcoholism' 'Alcohol Abuse' 'Screening and Brief Interventions']	Chemically-Induced Disorders	Multiple
**NCT03905863**	['Diabetic Foot Ulcer' 'Surgical Wound']	Cardiovascular Diseases	Multiple
**NCT05378399**	['HIV Infections' 'Substance Use' 'Adherence, Medication' 'Adherence, Treatment']	Behavior and Behavior Mechanisms	Multiple
**NCT05130112**	['Small Airway Disorders' 'COPD']	Pathological Conditions, Signs and Symptoms	Multiple
**NCT04169282**	['Tracheobronchomalacia']	Musculoskeletal Diseases	Multiple
**NCT05473702**	['Heart Disease Chronic' 'Pulmonary Disease, Chronic Obstructive' 'Blood Pressure' 'Heart Rhythm Disorder']	Respiratory Tract Diseases	Multiple
**NCT05212363**	['Compression; Vein' 'Compression; Artery' 'Sedentary Behavior' 'DVT of Legs']	Behavior and Behavior Mechanisms	Multiple

Table S3: Studies with conditions not mapped to MeSH headings by SciSpacy EntityLinker.

Clinical Trial	Study Official Title	Condition
**NCT04887922**	The Effect of Preoperative and Postoperative Incentive Spirometry in Patients Undergoing Major Abdominal Surgery	Abdominal Surgery
**NCT04910139**	A User Study of the Soniflow System for Nasal Congestion Relief	Nasal Congestion
**NCT02091882**	OSMITTER 316-13-206A Substudy: A Substudy to Measure the Accuracy of Ingestible Event Marker (IEM) Detection by the Medical Information Device #1 (MIND1) System and Determine the Latency Period	Device Latency
**NCT05052281**	Promoting Healthy Brain Development Via Prenatal Stress Reduction: An Innovative Precision Medicine Approach	Prenatal Stress
**NCT05099614**	Naloxone Administration Via Auto -injection in Healthy Volunteers	Overdose Antidote
**NCT05199844**	Accuracy of Apple Watch to Measure Cardiovascular Indices in Patients With Cardiac Diseases: Observational Study	Apple Watch

Table S4: Keyword components comprising each inclusion criteria topic.

Topic name	MeSH Category	Proportion	Components
**Clinical factors**	Pathological Conditions, Signs and Symptoms	21/38 (55·3%)	pain, months, sleep, scale, 10
	Nervous System Diseases	31/66 (47·0%)	pain, score, month, months, insomnia
	Mental Disorders	11/24 (45·8%)	treatment, medication, score, stable, month
	Nutritional and Metabolic Diseases	15/38 (39·5%)	smbg, participants, months, prior, therapy
	Cardiovascular Diseases	8/24 (33·3%)	score, states, resident, vasc, patients
	Neoplasms	5/17 (29·4%)	treatment, 20th, percentile, systemic, having
	Behavior and Behavior Mechanisms	9/32 (28·1%)	pain, usa, average, baseline, reported
**Informed consent**	Nutritional and Metabolic Diseases	24/38 (63·2%)	consent, informed, willing, provide, hipaa
	Behavior and Behavior Mechanisms	18/32 (56·2%)	consent, informed, provide, willing, required
	Neoplasms	9/17 (52·9%)	consent, informed, written, provide, willing
	Nervous System Diseases	32/66 (48·5%)	consent, informed, provide, willing, able
	Pathological Conditions, Signs and Symptoms	16/38 (42·1%)	consent, informed, provide, willing, signed
	Mental Disorders	9/24 (37·5%)	consent, informed, provide, willing, able
	Cardiovascular Diseases	6/24 (25·0%)	informed, consent, ascertained, able, valid
**Age (>18)**	Behavior and Behavior Mechanisms	23/32 (71·9%)	18, years, age, old, ages
	Nutritional and Metabolic Diseases	25/38 (65·8%)	years, age, 18, old, male
	Pathological Conditions, Signs and Symptoms	25/38 (65·8%)	18, years, age, aged, old
	Cardiovascular Diseases	14/24 (58·3%)	18, years, age, older, male
	Nervous System Diseases	38/66 (57·6%)	years, age, 18, old, aged
	Mental Disorders	13/24 (54·2%)	years, 18, age, 22, older
	Neoplasms	6/17 (35·3%)	years, 18, age, old, mole
**Smartphone access**	Nutritional and Metabolic Diseases	18/38 (47·4%)	smartphone, device, mobile, phone, compatible
	Neoplasms	8/17 (47·1%)	smartphone, access, recorded, audio, mobile
	Behavior and Behavior Mechanisms	12/32 (37·5%)	smartphone, android, access, iphone, ios
	Mental Disorders	7/24 (29·2%)	smartphone, iphone, game, android, controller
	Cardiovascular Diseases	6/24 (25·0%)	smartphone, plan, data, wi, fi
	Nervous System Diseases	11/66 (16·7%)	smartphone, access, device, holds, license
	Pathological Conditions, Signs and Symptoms	2/38 (5·3%)	comfortable, complete, zoom, conferencing, web
**English fluency**	Behavior and Behavior Mechanisms	11/32 (34·4%)	english, fluency, read, speaking, literacy
	Nutritional and Metabolic Diseases	12/38 (31·6%)	english, read, speaking, speak, write
	Neoplasms	5/17 (29·4%)	english, read, speak, speaking, write
	Pathological Conditions, Signs and Symptoms	10/38 (26·3%)	english, speaking, read, understand, spanish
	Cardiovascular Diseases	6/24 (25·0%)	english, speaking, read, write, speak
	Mental Disorders	6/24 (25·0%)	english, speaking, proficient, read, language
	Nervous System Diseases	15/66 (22·7%)	english, speaking, read, spanish, speak

Table S5: Keyword components comprising each exclusion criteria topic.

Topic name	MeSH Category	Proportion	Components
**Medical history**	Mental Disorders	23/24 (95·8%)	disorder, suicidal, use, current, months
	Pathological Conditions, Signs and Symptoms	33/38 (86·8%)	disorder, history, severe, current, use
	Cardiovascular Diseases	20/24 (83·3%)	heart, weeks, patients, cardiac, disorder
	Nervous System Diseases	55/66 (83·3%)	disorder, history, severe, disease, months
	Nutritional and Metabolic Diseases	31/38 (81·6%)	insulin, disease, disorder, months, investigator
	Behavior and Behavior Mechanisms	26/32 (81·2%)	disorder, current, smoking, suicidal, treatment
	Neoplasms	13/17 (76·5%)	disorder, history, care, therapy, psychiatric
**Pregnancy**	Nutritional and Metabolic Diseases	21/38 (55·3%)	pregnant, pregnancy, women, female, feeding
	Pathological Conditions, Signs and Symptoms	12/38 (31·6%)	pregnant, pregnancy, teeth, women, face
	Behavior and Behavior Mechanisms	9/32 (28·1%)	pregnant, planning, face, pregnancy, comfortable
	Nervous System Diseases	18/66 (27·3%)	pregnant, pregnancy, women, breastfeeding, potential
	Neoplasms	4/17 (23·5%)	pregnant, adults, populations, vulnerable, prisoners
	Mental Disorders	5/24 (20·8%)	pregnant, pregnancy, teenagers, cycles, menstrual
	Cardiovascular Diseases	3/24 (12·5%)	postpartum, wfbmc, center, location, birth
**Allergies or other skin conditions**	Neoplasms	5/17 (29·4%)	cancer, documented, skin, patients, hematologic
	Nutritional and Metabolic Diseases	11/38 (28·9%)	skin, cell, allergy, basal, neoplasms
	Pathological Conditions, Signs and Symptoms	7/38 (18·4%)	skin, cancer, carcinoma, sores, hardware
	Behavior and Behavior Mechanisms	4/32 (12·5%)	skin, fragile, dermatologic, intact, oozing
	Cardiovascular Diseases	3/24 (12·5%)	wound, patches, surface, adhesive, skin
	Mental Disorders	2/24 (8·3%)	cancers, skin, cvd, preexisting, angiomas
	Nervous System Diseases	5/66 (7·6%)	allergic, skin, known, tapes, reaction
**Cardiovascular metrics**	Cardiovascular Diseases	8/24 (33·3%)	mmhg, diastolic, cm, baseline, dl
	Nutritional and Metabolic Diseases	9/38 (23·7%)	mmhg, pressure, 60, ml, min
	Pathological Conditions, Signs and Symptoms	4/38 (10·5%)	inches, 79, clots, obesity, circumference
	Nervous System Diseases	5/66 (7·6%)	mmhg, 60, diastolic, 30, cm
	Neoplasms	1/17 (5·9%)	mass, 35, bmi, index, body
	Mental Disorders	1/24 (4·2%)	tsh, thyroid, pcp, mu, values
**Ability to provide informed consent**	Neoplasms	5/17 (29·4%)	consent, informed, sign, willing, inability
	Cardiovascular Diseases	3/24 (12·5%)	consent, provide, informed, inability, unwilling
	Nervous System Diseases	8/66 (12·1%)	consent, informed, provide, written, unable
	Nutritional and Metabolic Diseases	4/38 (10·5%)	consent, informed, inability, provide, unwillingness
	Pathological Conditions, Signs and Symptoms	3/38 (7·9%)	consent, informed, provide, inability, unable
	Behavior and Behavior Mechanisms	2/32 (6·2%)	informed, consent, inability, provide, unwillingness
	Mental Disorders	1/24 (4·2%)	consent, informed, adults, unable, written

Table S6: Incorrect values from manual assessment of topic modeling in a subset of 200 inclusion eligibility criteria.

NCTId	Inclusion Criteria (Preprocessed)	BERTopic cluster	Expected cluster
**NCT03142932**	agree anticipate living baltimore 2 months	Clinical factors	Other
**NCT04380415**	u.s. resident	Clinical factors	Other
**NCT03214224**	1	Clinical factors	NA
**NCT03418129**	served military branches army navy marines air force coast guard october 2001.	Clinical factors	Other
**NCT03335800**	current resident united states time eligibility screening defined self reported state residence 50 states united states district columbia.	Clinical factors	Other
**NCT04524598**	residing usa duration 5 week	Clinical factors	Other
**NCT04268914**	4. children normal range development recruited study. assessed report parents. rationale excluding patients developmental delay cognitive impairments children react stressors surgery differently children developmental delay. unclear children use preparation programs interventions included likely responses baseline outcome measures differ children normal developmental parameters.	Clinical factors	Ability to provide informed consent
**NCT04253691**	unable complete forms implement treatment cognitive impairment mmse<26	Clinical factors	Ability to provide informed consent
**NCT04607460**	able speak understand english 6 access computer tablet home email address.	Ability to provide informed consent	English fluency
**NCT03335800**	valid phone number associated iphone ascertained self report.	Ability to provide informed consent	Smartphone access
**NCT03338036**	holds valid driver s license	Smartphone access	Other

Table S7: Incorrect values from manual assessment of topic modeling in a subset of 200 exclusion eligibility criteria.

NCTId	Inclusion Criteria (Preprocessed)	BERTopic cluster	Expected cluster
**NCT05263037**	members household	Medical history	Other
**NCT04394754**	incarceration	Medical history	Other
**NCT03528174**	hematocrit 36 men 32 women.	Pregnancy	Cardiovascular metrics
**NCT04479735**	refuses lidocaine 2.5 prilocaine 2.5 cream use excluded study.	Medical history	Ability to provide informed consent
**NCT05293275**	injury eyes face neck impedes comfortable use virtual reality	Medical history	Medical history
**NCT04797611**	agree use approved contraception method entirety trial	Medical history	Ability to provide informed consent
**NCT05263037**	injury eyes face neck prevents comfortable use vr.	Pregnancy	Ability to provide informed consent
**NCT04906603**	loss consciousness greater 30 minutes	Cardiovascular metrics	Medical history
**NCT04230486**	volunteers unable complete tasks understand instructions	Medical history	Ability to provide informed consent
**NCT05112432**	legal commitment treatment medical guardianship provision guardianship order court order allow guardian consent research	Medical history	Ability to provide informed consent
**NCT03315286**	employee direct relative employee investigational site sponsor	Medical history	Ability to provide informed consent
**NCT04152447**	injuries requiring staged surgical fixation i.e. ex fix orif	Allergies or other skin conditions	Medical history
**NCT03996954**	patients unable unwilling use device	Medical history	Ability to provide informed consent

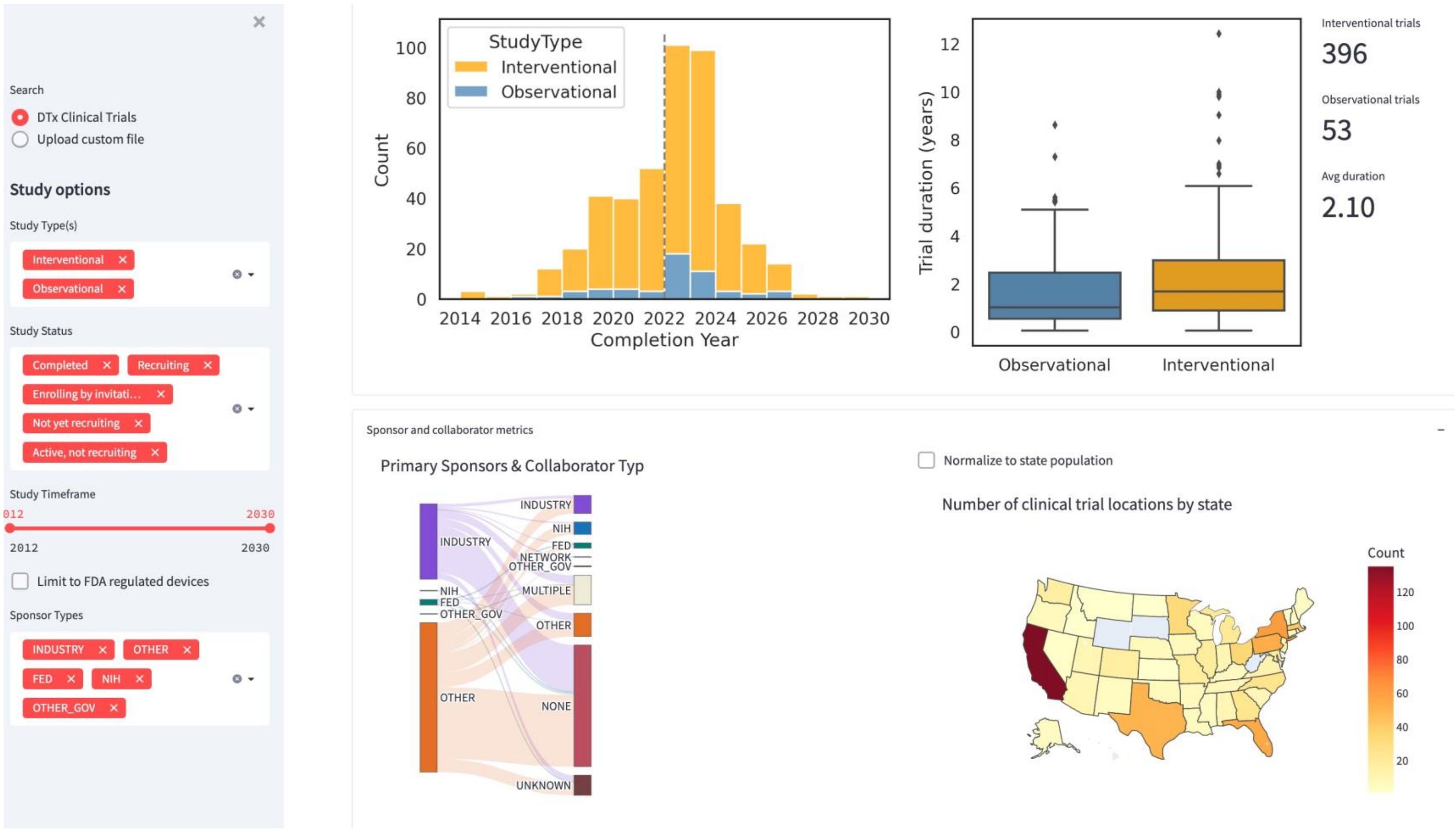

Figure S4. Clinical trials analysis dashboard. Screenshot of an interactive dashboard for analysis of ClinicalTrials.gov metadata for DTx clinical trials, provided for readers.
